# First Report of Domoic Acid Production from *Pseudo-nitzschia multistriata* in Paracas Bay (Peru)

**DOI:** 10.3390/toxins13060408

**Published:** 2021-06-09

**Authors:** Cecil Tenorio, Gonzalo Álvarez, Sonia Quijano-Scheggia, Melissa Perez-Alania, Natalia Arakaki, Michael Araya, Francisco Álvarez, Juan Blanco, Eduardo Uribe

**Affiliations:** 1 Banco de Germoplasma de Organismos Acuáticos, Instituto del Mar del Perú, Callao 07021, Peru; narakaki@imarpe.gob.pe; 2 Doctorado en Acuicultura, Programa Cooperativo Universidad de Chile, Pontificia Universidad Católica de Valparaíso, Coquimbo 17811421, Chile; euribe@ucn.cl; 3 Facultad de Ciencias del Mar, Departamento de Acuicultura, Universidad Católica del Norte, Coquimbo 1281, Chile; falvarezsego@gmail.com; 4 Centro de Investigación y Desarrollo Tecnológico en Algas (CIDTA), Facultad de Ciencias del Mar, Universidad Católica del Norte, Coquimbo 1281, Chile; mmaraya@ucn.cl; 5 Centro Universitario de Investigaciones Oceanológicas, Universidad de Colima, Manzanillo 28218, Mexico; quijano@ucol.mx; 6 Facultad de Ciencias, Universidad Nacional Agraria La Molina, Avenida La Universidad s/n, La Molina, Lima 15024, Peru; mlperal17@gmail.com; 7 Centro de Investigacións Mariñas (Xunta de Galicia), 36620 Vilanova de Arousa, Pontevedra, Spain; juan.carlos.blanco.perez@xunta.gal

**Keywords:** harmful algae, amnesic shellfish poisoning, ITS2, *Argopecten purpuratus*, scallop

## Abstract

The Peruvian sea is one of the most productive ecosystems in the world. Phytoplankton production provides food for fish, mammals, mollusks and birds. This trophic network is affected by the presence of toxic phytoplankton species. In July 2017, samples of phytoplankton were obtained from Paracas Bay, an important zone for scallop (*Argopecten purpuratus*) aquaculture in Peru. Morphological analysis revealed the presence of the genus *Pseudo-nitzschia*, which was isolated and cultivated in laboratory conditions. Subsequently, the monoclonal cultures were observed by scanning electron microscopy (SEM), and identified as *P. multistriata*, based on both the morphological characteristics, and internal transcribed spacers region (ITS2) sequence phylogenetic analysis. Toxin analysis using liquid chromatography (LC) with high-resolution mass spectrometry (HRMS) revealed the presence of domoic acid (DA) with an estimated amount of 0.004 to 0.010 pg cell^−1^. This is the first report of DA from the coastal waters of Peru and its detection in *P. multistriata* indicates that it is a potential risk. Based on our results, routine monitoring of this genus should be considered in order to ensure public health.

## 1. Introduction

Marine planktonic diatoms of the genus *Pseudo-nitzschia* are found in polar, warm and tropical regions; most of their species are cosmopolitan [1,2,3]. Currently, 56 species [4,5] of this genus have been reported, 26 of them producing the neurotoxic compound domoic acid (DA) [6]. Examination of the morphology by optical microscopy is frequently inconclusive, and for this reason scanning and/or transmission electron microscopy is also required. Additionally, molecular analysis of ITS2 [7] of the nuclear encoded ribosomal DNA can identify the species at the molecular level and differentiate between cryptic and pseudo-cryptic species [2,8,9,10,11,12].

The first intoxication in humans by DA was reported at Prince Edward Island, Canada, in 1987. More than 100 people reported becoming ill and at least three people died after eating blue mussels (*Mytilus edulis*) [13]. Digestive problems and short-term memory loss were the main symptoms of this intoxication, these led to the syndrome being named amnesic shellfish poisoning (ASP) [13,14,15]. Since the report of the first outbreak detected in Canada produced by the diatom *Pseudo-nitzschia multiseries*, episodes of DA have been recorded in many areas around the world [2,3]. Besides its effects on humans, DA also has severe effects on the trophic transfer between harmful microalgae, filter feeders and mollusks [16,17,18,19,20], spreading to fish [21] and causing mortality of birds and marine mammals [2,22,23,24].

Blooms of *Pseudo-nitzschia* spp., which produce DA, generate large economic losses due to long periods of closures for recreational and commercial fisheries or marketing of aquaculture products. Recently, these toxic outbreaks have been reported in the United States from 2015 to 2016, affecting the fisheries of the razor clam (*Siliqua patula*), Dungeness crab (*Metacarcinus magister*) and rock crabs (*Cancer productus*, *Metacarcinus anthonyi* and *Romaleon antennarium*) [3,24,25,26]. Likewise, in Europe, the DA-producing diatoms of genus *Pseudo-nitzschia* have led to bans on harvesting the natural populations of the king scallop *Pecten maximus*, and to the discouragement of aquaculture efforts for this species [27,28], due to its high capability to retain the toxin [29,30].

The coastal upwelling system of Peru constitutes a large part of the Humboldt Current system and is considered one of the most productive regions in the world, fixing 3000–4000 mg C m^−2^ d^−1^ [31,32,33,34]. Due to this high productivity, the area is susceptible to harmful algal blooms [2,35,36]. For Peruvian oceanic and coastal areas, the first report is from Hasle [37], who described the presence of *Pseudo-nitzschia pungens* (Grunow ex Cleve) Hasle and *P. australis* Frenguelli (as *P. pseudo seriata* G.R. Hasle) [2,37]. More recently, Tenorio, et al. [38] reported the presence of a non-toxic strain of *P. subpacifica* (Hasle) Hasle on the central coast of Peru, between San Lorenzo Island, Callao (12°03′S), and Paracas Bay, Ica (13°49′S). In northern Chile, within the framework of the Molluscan Shellfish Safety Program of the National Fisheries and Aquaculture Service (SERNAPESCA), elevated levels of DA have been detected in shellfish from many of the primary scallop *Argopecten purpuratus* aquaculture sites [39]. The blooms associated with those events have been dominated by diatom *Pseudo-nitzschia australis* with densities around 1.6 × 10^6^ cell L^−1^ [40].

In Peru, during the period 2011–2012, intoxication of fur seals (*Arctocephalus australis*) and sea lions (*Otaria byronia*) was reported in San Juan de Marcona, Ica (15°20’S), associated with the detection of DA in feces of these marine mammals. During this episode, *Pseudo-nitzschia* spp. were detected with a maximum of 88,580 cell L^−1^ in Paracas Bay sampling station (distance of ~155 km to the north) [41]. Unfortunately, there is no additional information about the species that formed the *Pseudo-nitzschia* assemblage.

Paracas Bay is a traditional aquaculture area of the scallop *A. purpuratus*, the most important bivalve species in Peru [42]. To date, within the framework of the Molluscan Shellfish Control Program run by the National Fisheries Health Organization of Peru (SANIPES), there is no information about the presence of DA in this bivalve. Nevertheless, the detection of DA in marine mammals in nearby areas indicates that the toxin is a potential risk to aquaculture and suggests that more research is necessary in order to identify different *Pseudo-nitzschia* species and their capability to produce DA on the coast of Peru.

In 2017, phytoplankton were collected in Paracas Bay to establish monoclonal cultures of *Pseudo-nitzschia* spp., for their morphological, molecular and toxicological characterization, in order to understand the potentially toxic species of the genus *Pseudo-nitzschia* in Peruvian waters.

## 2. Results

### 2.1. Morphological Analysis

**Figure 1:**
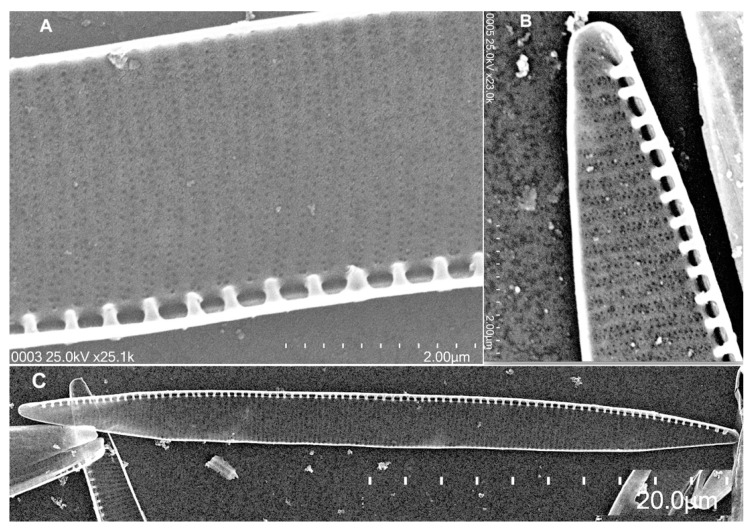
*Pseudo-nitzschia multistriata* (MEB), (**A**) whole valve; (**B**) valve end (2 µm); (**C**) mid valve, no central interspace (5 µm).

The isolated strain was morphologically identified as *Pseudo-nitzschia multistriata* (H. Takano) H. Takano. The cells of strain IMP-BG 440 (Figure 1) had a sigmoid shape in girdle view, and formed stepped chains, with up to four in length and an overlap of 1/6 of the total cell length. The cells were symmetrical and broad lanceolate in valve view. The apical axis ranged from 40 to 48 µm, while the transapical axis of the valves ranged from 3.20 to 4.30 µm. A large central interspace and a central nodule were absent. Valves contained 24 to 28 fibulae and 40 to 42 striae per 10 μm. Striae were formed by 2–3 rows of poroids with a density between 8 to 13 per 1 μm.

### 2.2. Molecular Analysis

**Figure 2:**
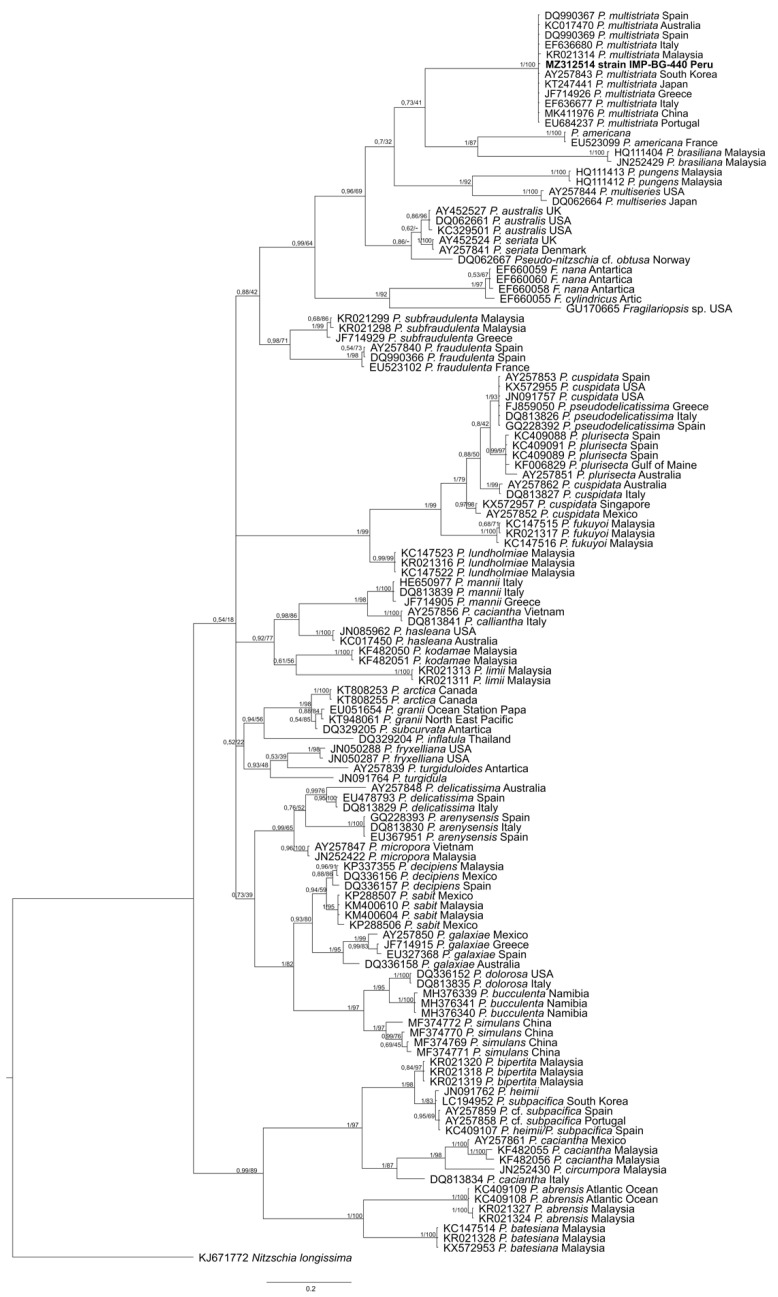
*Pseudo-nitzschia* Bayesian tree based on ITS2 sequences. Numbers above lines represent BI posterior probabilities/ML bootstrap values. “-“ indicates a different phylogeny structure for ML analysis. Boldface indicates the studied strain as *P. multistriata*. Phylogenetic ITS2 trees (BI and ML) showed six general groups. The taxon *P. multistriata* is included in one of these groups comprising also *P. americana* + *P. brasiliana*, *P. pungens* + *P. multiseries*, *P. australis* + *P.* seriata + *P.* cf. *obtusa*, *Fragilariopsis nana* + *F. cylindricus* + *Fragilariopsis* sp. and *P. subfraudulenta* + *P. fraudulenta*.

Final alignment yielded 414 characters and comprised 128 ITS2 sequences, including a short sequence (MZ312514, 132 pb) of the strain IMP-BG 440. Phylogenetic analysis of ITS2 sequences using maximum likelihood (ML) and Bayesian inference (BI) showed congruent topologies (Figure 2). Within the genus *Pseudo-nitzschia*, a well-supported monophyletic clade (BI/ML, 1/100) corresponded to *P*. *multistriata*, and 11 strains from Australia, Malaysia, Taiwan, Japan, South Korea, China, Greece, Italy, Spain and Portugal. The clade containing *P. multistriata* also contained species of *Pseudo-nitzschia* from France (*P. americana*) and Malaysia (*P. braziliana*) with low support (BI, 0.73). This *Pseudo-nitzschia* clade was positioned within a larger unsupported clade (BI, 0.70) containing *Pseudo-nitzschia* species from Malaysia (genera type *P. pungens*), Japan and the USA (*P. multiseries*). Additionally, the phylogenetic tree shows that *P. multistriata* is grouped with other species of *Pseudo-nitzschia* with different levels of support. However, five sequences of *Fragilariopsis* from Artic, Antarctica and the USA form a supported clade within species of *Pseudo-nitzschia* (BI/ML, 0.99/64).

### 2.3. Toxin Analysis

**Figure 3:**
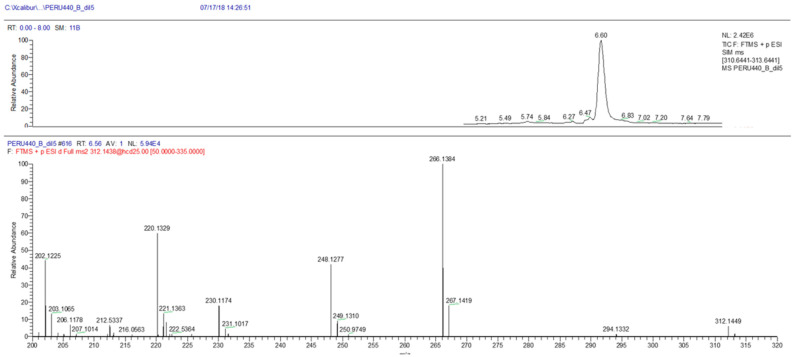
SIM chromatogram (upper panel) and mass spectrum (lower panel) of DA in *Pseudo-nitzschia multistriata* culture.

Analysis of extracts (*n* = 3) of the strain IMP-BG 440 showed that it contained domoic acid (Figure 3). Toxin analysis by LC-HRMS showed a chromatographic peak with a retention time of 6.60 min corresponding to the ion [M+H]^+^ 312.1449 *m/z* (mass deviation: 0.64 ppm). The fragmentation mass spectrum of the ion [M+H]^+^ 312.1449 *m/z* confirmed the identification of domoic acid (DA) because of its characteristic fragment MS/MS at 294.1332, 266.1384, 248.1277 and 220.1329 *m/z* (mass deviation in Table S1; Supplementary Materials). The estimated amount was between 0.004 to 0.010 pg cell^−1^.

## 3. Discussion

The species of the genus *Pseudo-nitzschia* are distributed throughout all the coasts of the world [1,2,3]. The present study provides the first report of *Pseudo-nitzschia multistriata* in Peruvian coastal waters and, as far as we know, in the southeastern Pacific area. The presence of this species has been reported from different geographical locations around the world (Table S2, Supplementary Materials).

The morphological examination of *Pseudo-nitzschia* cells (Table S2, Supplementary Materials) from the obtained cultures agrees in length of apical axis with the description of strains of *P. multistriata* from China [43], Tunisia [44], Catalan Coast [45,46], Gulf of Naples [47,48] and the Western Adriatic Sea [49]. Similarly, the width of the transapical axis corresponds to descriptions of cells from Ria de Aveiro, Portugal [50], Tokyo bay [51], New Zealand [52], Mexico [53] and Uruguay [54]. However, the length of the apical axis and the width of the transapical axis of the cell do not match the first description made by Takano [55], given that those were smaller.

The number of fibulae of Paracas strains was close to descriptions of cells from Ria de Aveiro, Portugal [50], Greek coastal waters [56], Gulf Naples, Italy [57] and Gulf of Trieste, Northern Adriatic Sea, Italy [58]. Finally, the striae were formed by 2–3 rows of poroids and their density were similar to those described in cells from Fukukoka Bay, Japan [55] and the Sea of Japan [59].

Phylogenetic analysis of ITS2 sequences support the morphological identification of the strain isolated from Paracas Bay as *P. multistriata*. This strain from Peru is situated with *P. multistriata* strains from Australia, Malaysia, South Korea, Taiwan, Japan, China and Europe, forming a well-supported monophyletic clade (BI/ML, 1/100). The phylogenetic tree also shows that *P. multistriata* is grouped with *P. brasiliana* and *P. americana*, within a clade comprising *P. pungens* (genera type), *P. multiseries*, *P. australis*, *P. seriata* and *P. obtuse*. Previous phylogenetic analyses of ITS2 sequences had pointed to different relationships of *P. multistriata* with *P. americana*, *P. brasiliana*, and *P. pungens*. Huang, et al. [60] showed that *P. americana* is placed on the base of the clade, not clustered with *P. multistriata* and *P. brasiliana*. On the contrary, Lim, et al. [61] showed that *P. multiseries* and *P. pungens* are placed at the base of the tree. Morphological characteristics have been included by Lim, et al. [61] as representative of some species of the *Pseudo-nitzschia* clade. Thus, morphological characters, 2–4 rows of poroids, the absence of a central nodule and the lower number of fibulae versus striae in 10 µm, were observed in *P. multistriata* from Japan [62], matching the description of the strain of *P. multistriata* (IMP-BG 440) from Peru.

This study confirms *P. multistriata* as an unequivocal source of domoic acid (DA) on the coast of Peru. The strain IMP-BG 440 tested was able to produce the toxin in culture with a concentration between 0.004 and 0.010 pg cell^−1^, which is comparable to those reported by Pistocchi, et al. [49] (0.003 pg cell^−1^) in a strain obtained from the Adriatic Sea that was cultured under similar conditions (16–18 °C; 60–100 μmol photons m^−2^ s^−1^). These concentrations were lower than the values reported in Australian strains by Ajani, et al. [63] (1–11 pg cell^−1^), Rhodes, et al. [64] (1.5 pg cell^−1^) and in Italian strains registered by Amato, et al. [65] (0.28 pg cell^−1^), Orsini, et al. [47] (0.69 pg cell^−1^) and Sarno [48] (0.65 pg cell^−1^).

The Humboldt Current system (HCS) is considered one of the most productive fishery regions in the world oceans [33,34,66,67]. As mentioned above, due to its high productivity, this upwelling area is susceptible to harmful algal blooms (HABs) [35,36]. In this context, other toxic *Pseudo-nitzschia* species have been reported in the HCS, specifically on the northern Chilean coast [68,69]. In some cases, DA concentrations have exceeded the regulatory limit (20 mg·kg^−1^) and the harvesting of scallops (*A. purpuratus*), from aquaculture sites, has therefore been banned [40]. The DA content in *P. multistriata* (strain IMP-BG 440) was substantially lower than those reported in *P. australis* (1.74 pg cell^−1^) for the southeastern Pacific; however, it was close to the value of *P. calliantha* (0.01 pg cell^−1^) [39]. The low content of DA in *P. multistriata* in Peruvian waters could be one of the reasons that there has not been any detection of this toxin in scallops cultivated in Paracas Bay in the framework of the Molluscan Shellfish Control Program run by SANIPES. A second reason could be the rapid DA depuration of this bivalve in the natural environment as has been demonstrated by Álvarez, et al. [70] in scallops cultivated in Tongoy Bay, Chile. However, the information provided by this work should be taken into consideration in the development of the Molluscan Shellfish Control Program ran by SANIPES [71].

Regarding the intoxication of marine mammals with low levels of DA on the Peruvian coast [41], it is clear that *P. multistriata* could be involved. However, with the available information we cannot discard the possibility that other species of *Pseudo-nitzschia* or more toxic strains than the one found in this study could be the principal cause of pinniped intoxication. Finally, more research is needed to find other toxic species, as well as the roll of different environmental variables in the production of DA in different strains of *P. multistriata* obtained along the Peruvian coast.

## 4. Conclusions

*Pseudo-nitzschia multistriata* has been identified from the Peruvian coast based on morphological, phylogenetic and molecular evidence. This is the first report of this species for the Southeast Pacific. The species is confirmed to be a producer of DA which makes it the first known DA producer from Peruvian waters. The presence of toxic *P. multistriata* is a potential risk for mammals, making it necessary to routinely monitor this species in order to protect public health, as well as the ecosystem of Paracas Bay.

## 5. Materials and Methods

### 5.1. Biological Samples and Establishment of Cultures

**Figure 4:**
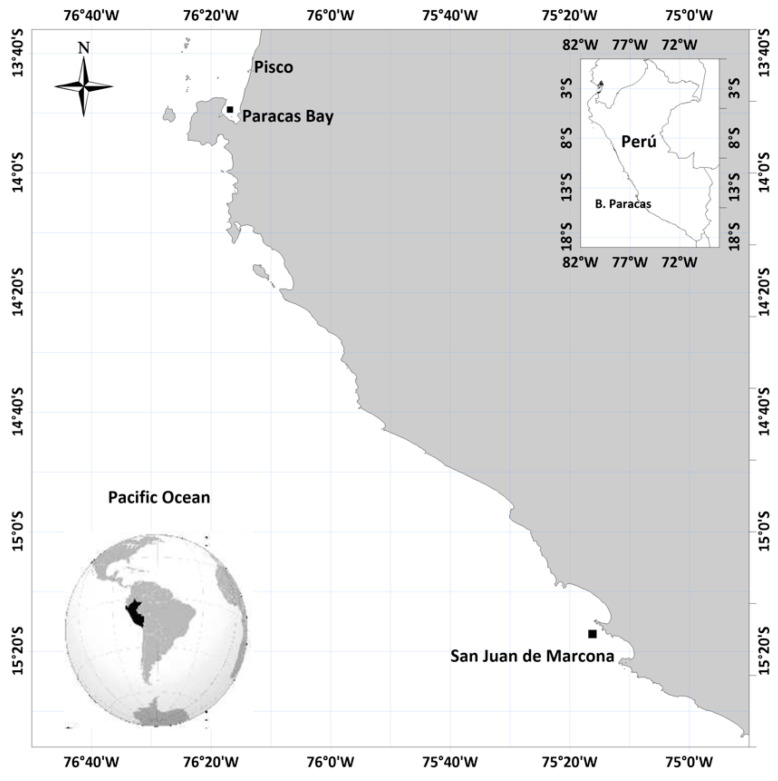
Location of the sampling station Paracas Bay, Peru.

Phytoplankton samples were obtained periodically in August 2017 in Paracas Bay (13°49′S, 76°17′O) (Figure 4) with temperatures of around 15 to 17 °C and salinity of 35.

Samples were collected using vertical net hauls (20 μm mesh), stored in 250 mL glass bottles and transported to the laboratory in the dark and chilled on ice (10 °C). To establish cultures of the *Pseudo-nitzschia* species, single chains of *Pseudo-nitzschia* cells were picked by micropipette and transferred to multi-well culture plates (hydrobios, Germany) filled with 2 mL of L1 culture medium [72] with a salinity of 30. The plates were maintained at 15 °C in a 12:12-h light: dark cycle, with a photon flux of 60 μmol photons m^−2^ s^−1^. Established cultures were transferred to borosilicate Erlenmeyer flasks with 150 mL of f/2 medium and grown at 15 °C in a 12:12-h light: dark cycle, with a photon flux of 80 μmol photons m^−2^ s^−1^. Mass cultures were grown in borosilicate bottles with 1 L of f/2 medium in triplicate under the above conditions. Two milliliter aliquots of the cultures were preserved with Lugol’s solution for the direct count of the cells. The cell densities in the samples were quantified by the Utermöhl method described by Hasle [73].

### 5.2. Morphological Analysis

Scanning electron microscopy (SEM) was used to perform detailed morphological analyses of the *Pseudo-nitzschia* cells. Organic matter was removed from the frustules following the methodology described by Lundholm, et al. [74]. The clean material was retained on a 5.0 μm membrane filter (Isopore Merck KGaA, Darmstadt, Germany), and washed with distilled water to remove salts and preservatives. After being airdried overnight, specimens were gold-coated in a JEOL JFC-1100 (JEOL Ltd., Tokyo, Japan) and observed with a Hitachi SU3500 scanning electron microscope (Hitachi High-Technologies Corporation, Tokyo, Japan). *Pseudo-nitzschia* cells were examined for morphometric characteristics that included width and length of the valve, density of striae, fibulae and poroids.

### 5.3. Molecular and Phylogenetic Analysis

Molecular identification of the strain of the *Pseudo-nitzschia* genus was performed by analyzing sequences of the internal transcribed spacer two (ITS2) region. When initial cultures of the strain reached the exponential growth phase, cells were concentrated by successive centrifugations and frozen at −80 °C prior to DNA extraction (24 h). Total genomic DNA was extracted following the cetyltrimethylammonium bromide (CTAB) method [75]. Part of the internal transcribed spacer (ITS) region was amplified by PCR, using ITS1/ITS4 primers (BIOSEARCH TECHNOLOGIES, Petaluma, CA, USA) [76]. The polymerase chain reaction (PCR) conditions for ITS include pre denaturation at 95 °C for 3 min, followed by 39 cycles of 95 °C for 30 s, 45 °C for 30 s and 72 °C for 50 s; and finally, 71 °C for 7 min. The amplicons were visualized on agarose gel (1.2%) (Invitrogen, Carlsbad, USA), purified and sequenced for one strand by Macrogen Inc. (South Korea).

Sequences obtained were checked in BIOEDIT v.7.0.5.3 (Raleigh, NC, USA., 2005) [77] and compared in the GenBank public database using the Basic Local Alignment Search Tool BLASTn. The data block used in the molecular analyses consisted of 128 ITS sequences (Table S3), including the one sequence of *Pseudo-nitzschia* obtained in this study, sequences of *Pseudo-nitzschia* and 5 sequences of *Fragilariopsis* available in the public database and a sequence of *Nitzschia longissima* as an outgroup. The alignment was constructed using the Muscle algorithm in MEGA7v.7.0.26 (Philadelphia, PA, USA., 2016) [78], checked visually, corrected and trimmed using MEGA7 so that it only contained sequences of the ITS2 region. Final alignment was independently analyzed using maximum likelihood (ML) and Bayesian inference (BI). The best evolutionary models for ML and BI were calculated in jModelTest 2 (Spain, 2012) [79] using the Akaike information criterion (AIC) and the Bayesian information criterion (BIC), respectively. ML analysis was carried out in RAxML v.8.2.X (Karlsruhe, Germany, 2014) [80] using the graphic user interface raxmlGUI v.1.5 5b1 (Frankfurt, Germany, 2012) [81] with the selected model (GTR+I+G) and 1000 bootstrap replications. BI was carried out in MrBayes v.3.2.6 [82] with the selected model (HKY+I+G), two runs of 10 million Markov chain Monte Carlo generations each with 1 cold chain and 3 heated chains, sampling and printing every 1000 generations. The convergence of the runs was checked using Tracer v.1.6.0 (Edinburgh, UK, 2014). A consensus tree was constructed after a burn-in of 25%, and posterior probabilities were estimated.

### 5.4. Sample Preparation and Toxin Analysis

A 1 L sample from the culture of *Pseudo-nitzschia* spp. (densities ranged from 267,170 to 305,691 cell mL^−1^) was taken in the stationary phase of growth. The sample was concentrated by centrifugation at 4000 g for 10 min with a centrifuge (Hettich Rotina 420R, Germany). The obtained pellets were mixed with 10 mL of aqueous methanol (Merck KGaA, Darmstadt, Germany) (50%, *v/v*) and the cells disrupted with a Branson Ultrasonic 250 (Danbury, CT, USA). The extract was clarified by centrifugation at 10,000 g for 20 min (Centurion K2015R, Centurion Scientific Ltd., Stoughton, West Sussex, UK). A one-milliliter aliquot was filtered through 0.22 μm Clarinert nylon syringe filters (13 mm diameter) (Bonna-Agela technologies, Torrance, CA, USA) and stored in an autosampler vial at −20 °C until analysis. The presence of DA (cellular content) in the extracts was determined following the method described by de la Iglesia, et al. [83] with modifications. The instrumental analysis was developed using a Dionex Ultimate 3000 UHPLC system (Thermo Fisher Scientific, Sunnyvale, CA, USA). A reversed-phase HPLC column Kinetex C18 (50 mm × 2.1 mm; 2.6 µm) with an Ultra Guard column C18 from Phenomenex (Torrance, CA, USA) was used. The flow rate was set to 0.28 mL min^−1^, and the injection volume was 10 µL. Mobile phases A and B were water (Milli-Q) and MeOH, respectively, both containing 0.1% formic acid. The following gradient was used to achieve the chromatographic separation: 100% phase A, held for the first 0.5 min. Afterwards, separation was carried out at 12.5% B up to 3 min, decreased to 3% B over 7 min and then returned to the initial conditions over 2 min. The total analysis run time was 12 min.

The detection of DA was carried out by a high-resolution mass spectrometer Q Exactive Focus equipped with an electrospray interphase HESI II (Thermo Fisher Scientific, Sunnyvale, CA, USA). The interface was operated in positive ionization mode with a spray voltage of 3.5 kV. The temperature of the ion transfer tube and the HESI II vaporizer were set at 250 °C. Nitrogen (>99.98%) was employed as a sheath gas and auxiliary gas at pressures of 20 and 10 arbitrary units, respectively. Data were acquired in selected ion monitoring (SIM) and data-dependent (ddMS^2^) acquisition modes (for quantification and confirmation, respectively). In SIM mode, the mass was set to 312.1404 *m*/*z*, the scan mass range was set at *m*/*z* 100–1000 with a mass resolution of 70,000, the automatic gain control (AGC) was set at 5 × 10^4^ and the maximum injection time (IT) 3000 ms. For dds^2^ the mass resolution was set at 70,000, AGC at 5 × 10^4^ and IT 3000 ms. In both cases, the isolation windows were 2 *m*/*z*. DA was quantified by external calibration, using a DA-certified reference solution (CRM-DA-g) (NRC, CNRC, Canada). Limits of detection were calculated as the average concentration of DA producing a signal-to-noise ratio (S/N) of 3 and corresponding to 0.5 ng mL^−1^, while the limit of quantification of the method was 2 ng mL^−1^.

## Data Availability

Not applicable.

## Supplementary Materials

The following are available online at https://www.mdpi.com/article/10.3390/toxins13060408/s1, Table S1: Accurate mass and mass deviation (ppm) of domoic acid and its main fragments. Table S2: Comparison of morphometric data between Peruvian strain of *Pseudo-nitzschia multistriata* with strains obtained from different locations around the world, Table S3: List of sequences of *Pseudo-nitzschia*, *Fragilariopsis* and *Nitzschia* strains included in the molecular analysis. Species, locality, strain code and GenBank accession numbers for the ITS2 gene marker.

## References

[1] Hasle G.R. (2002). Are most of the domoic acid-producing species of the diatom genus Pseudo-nitzschia cosmopolites?. Harmful Algae.

[2] Trainer V.L., Bates S.S., Lundholm N., Thessen A.E., Cochlan W.P., Adams N.G., Trick C.G. (2012). Pseudo-nitzschia physiological ecology, phylogeny, toxicity, monitoring and impacts on ecosystem health. Harmful Algae.

[3] Bates S.S., Hubbard K.A., Lundholm N., Montresor M., Leaw C.P. (2018). Pseudo-nitzschia, Nitzschia, and domoic acid: New research since 2011. Harmful Algae.

[4] Chen X.M., Pang J.X., Huang C.X., Lundholm N., Teng S.T., Li A., Li Y. (2021). Two New and Nontoxigenic Pseudo-nitzschia species (Bacillariophyceae) from Chinese Southeast Coastal Waters. J. Phycol..

[5] Guiry M.D., Guiry G.M. AlgaeBase. http://www.algaebase.org.

[6] Lundholm N. Bacillariophyceae, in IOC-UNESCO Taxonomic Reference List of Harmful Micro Algae. http://www.marinespecies.org/hab.

[7] Coleman A.W. (2003). ITS2 is a double-edged tool for eukaryote evolutionary comparisons. Trends Genet..

[8] Quijano-Scheggia S.I., Garcés E., Lundholm N., Moestrup Ø., Andree K., Camp J. (2009). Morphology, physiology, molecular phylogeny and sexual compatibility of the cryptic Pseudo-nitzschia delicatissima complex (Bacillariophyta), including the description of P. arenysensis sp. nov. Phycologia.

[9] Quijano-Scheggia S.I., Olivos-Ortiz A., Garcia-Mendoza E., Sánchez-Bravo Y., Sosa-Avalos R., Salas Marias N., Lim H.C. (2020). Phylogenetic relationships of Pseudo-nitzschia subpacifica (Bacillariophyceae) from the Mexican Pacific, and its production of domoic acid in culture. PLoS ONE.

[10] Lundholm N., Moestrup Ø., Hasle G.R., Hoef-Emden K. (2003). A study of the Pseudonitzschia pseudodelicatissima/cuspidata complex (Bacillariophyceae): What is P. pseudodelicatissima?. J. Phycol..

[11] Lundholm N., Bates S.S., Baugh K.A., Bill B.D., Connell L.B., Léger C., Trainer V.L. (2012). Cryptic and pseudo-cryptic diversity in diatoms—with descriptions of pseudo-nitzschia hasleana sp. nov. and p. fryxelliana sp. nov. 1. J. Phycol..

[12] Amato A., Kooistra W.H., Ghiron J.H.L., Mann D.G., Pröschold T., Montresor M. (2007). Reproductive isolation among sympatric cryptic species in marine diatoms. Protist.

[13] Bates S., Bird C.J., Freitas A.d., Foxall R., Gilgan M., Hanic L.A., Johnson G.R., Mc Culloch A., Odense P., Pocklington R. (1989). Pennate diatom Nitzschia pungens as the primary source of domoic acid, a toxin in shellfish from eastern Prince Edward Island, Canada. Can. J. Fish. Aquat. Sci..

[14] Perl T.M., Bédard L., Kosatsky T., Hockin J.C., Todd E.C., Remis R.S. (1990). An outbreak of toxic encephalopathy caused by eating mussels contaminated with domoic acid. N Engl. J. Med..

[15] Wright J., Boyd R., Freitas A.d., Falk M., Foxall R., Jamieson W., Laycock M., McCulloch A., McInnes A., Odense P. (1989). Identification of domoic acid, a neuroexcitatory amino acid, in toxic mussels from eastern Prince Edward Island. Can. J. Chem..

[16] Bates S. (1997). Toxic phytoplankton on the Canadian east coast: Implications for aquaculture. Bull. Aquacult. Assoc. Can..

[17] La Barre S., Bates S.S., Quilliam M.A., La Barre S., Kornprobst J.M. (2014). Domoic acid. Outstanding Marine Molecules: Chemistry, Biology, Analysis.

[18] Goldberg J.D. (2003). Domoic Acid in the Benthic Food Web of Monterey Bay, California. Master’s Thesis.

[19] Zabaglo K., Chrapusta E., Bober B., Kaminski A., Adamski M., Bialczyk J. (2016). Environmental roles and biological activity of domoic acid: A review. Algal Res..

[20] Bates S.S., Garrison D.L., Horner R.A. (1998). Bloom dynamics and physiology of domoic-acid-producing Pseudo-nitzschia species. Nato Asi Ser. G Ecol. Sci..

[21] Lefebvre K.A., Frame E.R., Kendrick P.S. (2012). Domoic acid and fish behavior: A review. Harmful Algae.

[22] D’Agostino V.C., Degrati M., Sastre V., Santinelli N., Krock B., Krohn T., Dans S.L., Hoffmeyer M.S. (2017). Domoic acid in a marine pelagic food web: Exposure of southern right whales Eubalaena australis to domoic acid on the Peninsula Valdes calving ground, Argentina. Harmful Algae.

[23] Di Liberto T. (2015). This summer’s West Coast algal bloom was unusual. What would Usual Look Like?.

[24] McCabe R.M., Hickey B.M., Kudela R.M., Lefebvre K.A., Adams N.G., Bill B.D., Gulland F.M., Thomson R.E., Cochlan W.P., Trainer V.L. (2016). An unprecedented coastwide toxic algal bloom linked to anomalous ocean conditions. Geophys. Res. Lett..

[25] Ritzman J., Brodbeck A., Brostrom S., McGrew S., Dreyer S., Klinger T., Moore S.K. (2018). Economic and sociocultural impacts of fisheries closures in two fishing-dependent communities following the massive 2015 US West Coast harmful algal bloom. Harmful Algae.

[26] Du X., Peterson W., Fisher J., Hunter M., Peterson J. (2016). Initiation and development of a toxic and persistent Pseudo-nitzschia bloom off the Oregon coast in spring/summer 2015. PLoS ONE.

[27] Arévalo F., Bermúdez de la Puente M., Salgado C. (1997). Seguimiento de biotoxinas marinas en las Rías Gallegas: Control y evolución durante los años 1995–1996. V Reunión Ibérica de Fitoplancton Tóxico y Biotoxinas. ANFACO-CECOPESCA, Vigo.

[28] Blanco J., Acosta C., De La Puente M.B., Salgado C. (2002). Depuration and anatomical distribution of the amnesic shellfish poisoning (ASP) toxin domoic acid in the king scallop Pecten maximus. Aquat. Toxicol..

[29] Mauriz A., Blanco J. (2010). Distribution and linkage of domoic acid (amnesic shellfish poisoning toxins) in subcellular fractions of the digestive gland of the scallop Pecten maximus. Toxicon.

[30] Blanco J., Mauríz A., Álvarez G. (2020). Distribution of Domoic Acid in the Digestive Gland of the King Scallop Pecten maximus. Toxins.

[31] Calienes R., Guillén O., Lostaunau N. (1985). Variabilidad espacio-temporal de clorofila, producción primaria y nutrientes frente a la costa peruana. Boletin Instituto del Mar del Peru.

[32] Graco M., Ledesma J., Flores G., Giron M. (2007). Nutrients, oxygen and biogeochemical processes in the Humboldt upwelling current system off Peru. Rev. Peru. Biol.

[33] Echevin V., Gévaudan M., Espinoza-Morriberón D., Tam J., Aumont O., Gutierrez D., Colas F. (2020). Physical and biogeochemical impacts of RCP8. 5 scenario in the Peru upwelling system. Biogeosciences.

[34] Bakun A., Weeks S.J. (2008). The marine ecosystem off Peru: What are the secrets of its fishery productivity and what might its future hold?. Prog. Oceanogr..

[35] Pitcher G.C., Jiménez A.B., Kudela R.M., Reguera B. (2010). Harmful algal blooms in eastern boundary upwelling systems. Oceanography.

[36] Trainer V.L., Pitcher G.C., Reguera B., Smayda T.J. (2010). The distribution and impacts of harmful algal bloom species in eastern boundary upwelling systems. Prog. Oceanogr..

[37] Hasle G.R. (1965). Nitzschia and Fragilariopsis species studied in the light and electron microscopes. II. The group Pseudonitzschia. Skr. Nor. Vidensk-Akad. I. Mat.-Nat. Kl. Ny Ser..

[38] Tenorio C., Uribe E., Gil-Kodaka P., Blanco J., Álvarez G. (2016). Morphological and toxicological studies ofPseudo-nitzschiaspecies from the central coast of Peru. Diatom Res..

[39] Álvarez G., Uribe E., Quijano-Scheggia S., López-Rivera A., Mariño C., Blanco J. (2009). Domoic acid production by Pseudo-nitzschia australis and Pseudo-nitzschia calliantha isolated from North Chile. Harmful Algae.

[40] Díaz P.A., Álvarez A., Varela D., Pérez-Santos I., Díaz M., Molinet C., Seguel M., Aguilera-Belmonte A., Guzmán L., Uribe E. (2019). Impacts of harmful algal blooms on the aquaculture industry: Chile as a case study. Perspect. Phycol.

[41] Fire S.E., Adkesson M.J., Wang Z., Jankowski G., Cárdenas-Alayza S., Broadwater M. (2017). Peruvian fur seals (Arctocephalus australis ssp.) and South American sea lions (Otaria byronia) in Peru are exposed to the harmful algal toxins domoic acid and okadaic acid. Mar. Mammal Sci..

[42] Kluger L.C., Taylor M.H., Wolff M., Stotz W., Mendo J. (2019). From an open-access fishery to a regulated aquaculture business: The case of the most important Latin American bay scallop (Argopecten purpuratus). Rev. Aquac..

[43] Lü S., Li Y., Lundholm N., Ma Y., Ho K. (2012). Diversity, taxonomy and biogeographical distribution of the genus Pseudo-nitzschia (Bacillariophyceae) in Guangdong coastal waters, South China Sea. Nova Hedwig..

[44] Sahraoui I., Grami B., Bates S.S., Bouchouicha D., Chikhaoui M.A., Mabrouk H.H., Hlaili A.S. (2012). Response of potentially toxic Pseudo-nitzschia (Bacillariophyceae) populations and domoic acid to environmental conditions in a eutrophied, SW Mediterranean coastal lagoon (Tunisia). Estuar. Coast. Shelf Sci..

[45] Quijano-Scheggia S., Garcés E., Andree K.B., De la Iglesia P., Diogène J., Fortuño J.M., Camp J. (2010). Pseudo-nitzschia species on the Catalan coast: Characterization and contribution to the current knowledge of the distribution of this genus in the Mediterranean Sea. Sci. Mar..

[46] Quijano-Sheggia S., Garcés E., Sampedro N., Van Lenning K., Flo Arcas E., Andree K., Fortuño Alós J.M., Camp J. (2008). Identification and characterisation of the dominant Pseudo-nitzschia species (Bacillariophyceae) along the NE Spanish coast (Catalonia, NW Mediterranean). Sci. Mar..

[47] Orsini L., Sarno D., Procaccini G., Poletti R., Dahlmann J., Montresor M. (2002). Toxic Pseudo-nitzschia multistriata (Bacillariophyceae) from the Gulf of Naples: Morphology, toxin analysis and phylogenetic relationships with other Pseudo-nitzschia species. Eur. J. Phycol..

[48] Sarno D. (2000). Production of domoic acid in another species of Pseudo-nitzschia: P. multistriata in the Gulf of Naples (Mediterranean Sea). Harmful Algal News.

[49] Pistocchi R., Guerrini F., Pezzolesi L., Riccardi M., Vanucci S., Ciminiello P., Dell’Aversano C., Forino M., Fattorusso E., Tartaglione L. (2012). Toxin levels and profiles in microalgae from the North-Western Adriatic Sea—15 years of studies on cultured species. Mar. Drugs.

[50] Churro C.I., Carreira C.C., Rodrigues F.J., Craveiro S.C., Calado A.J., Casteleyn G., Lundholm N. (2009). Diversity and abundance of potentially toxic Pseudo-nitzschia Peragallo in Aveiro coastal lagoon, Portugal and description of a new variety, P. pungens var. aveirensis var. nov. Diatom Res..

[51] Yap-Dejeto L.G., Omura T., Nagahama Y., Fukuyo Y. (2010). Observations of eleven Pseudo nitzschia species in Tokyo Bay, Japan. La mer.

[52] Rhodes L.L., Adamson J., Scholin C. (2000). Pseudo-nitzschia multistriata(Bacillariophyceae) in New Zealand. New Zealand J. Mar. Freshw. Res..

[53] Rivera-Vilarelle M., Quijano-Scheggia S., Olivos-Ortiz A., Gaviño-Rodríguez J.H., Castro-Ochoa F., Reyes-Herrera A. (2013). The genus Pseudo-nitzschia (Bacillariophyceae) in Manzanillo and Santiago Bays, Colima, Mexico. Bot. Mar..

[54] Méndez S.M., Ferrario M., Cefarelli A.O. (2012). Description of toxigenic species of the genus Pseudo-nitzschia in coastal waters of Uruguay: Morphology and distribution. Harmful Algae.

[55] Takano H. (1993). Marine diatom Nitzschia multistriata sp. nov. common at inlets of southern Japan. Diatom.

[56] Moschandreou K.K., Baxevanis A.D., Katikou P., Papaefthimiou D., Nikolaidis G., Abatzopoulos T.J. (2012). Inter- and intra-specific diversity of Pseudo-nitzschia (Bacillariophyceae) in the northeastern Mediterranean. Eur. J. Phycol..

[57] D’Alelio D., Amato A., Kooistra W.H., Procaccini G., Casotti R., Montresor M. (2009). Internal transcribed spacer polymorphism in Pseudo-nitzschia multistriata (Bacillariophyceae) in the Gulf of Naples: Recent divergence or intraspecific hybridization?. Protist.

[58] Dermastia T.T., Cerino F., Stanković D., Francé J., Ramšak A., Tušek M.Ž., Beran A., Natali V., Cabrini M., Mozetič P. (2020). Ecological time series and integrative taxonomy unveil seasonality and diversity of the toxic diatom Pseudo-nitzschia H. Peragallo in the northern Adriatic Sea. Harmful Algae.

[59] Stonik I.V., Orlova T.Y., Lundholm N. (2011). Diversity of Pseudo-nitzschia H. Peragallo from the western North Pacific. Diatom Res..

[60] Huang C.X., Dong H.C., Lundholm N., Teng S.T., Zheng G.C., Tan Z.J., Lim P.T., Li Y. (2019). Species composition and toxicity of the genus Pseudo-nitzschia in Taiwan Strait, including P. chiniana sp. nov. and P. qiana sp. nov. Harmful Algae.

[61] Lim H.C., Tan S.N., Teng S.T., Lundholm N., Orive E., David H., Quijano-Scheggia S., Leong S.C.Y., Wolf M., Bates S.S. (2018). Phylogeny and species delineation in the marine diatom Pseudo-nitzschia (Bacillariophyta) using cox1, LSU, and ITS2 rRNA genes: A perspective in character evolution. J. Phycol..

[62] Stonik I., Orlova T.Y., Propp L., Demchenko N., Skriptsova A. (2012). An autumn bloom of diatoms of the genus Pseudo-nitzschia H. Peragallo, 1900 in Amursky Bay, the Sea of Japan. Russ. J. Mar. Biol..

[63] Ajani P., Murray S., Hallegraeff G., Lundholm N., Gillings M., Brett S., Armand L. (2013). The diatom genus Pseudo-nitzschia (Bacillariophyceae) in New South Wales, A ustralia: Morphotaxonomy, molecular phylogeny, toxicity, and distribution. J. Phycol..

[64] Rhodes L., Jiang W., Knight B., Adamson J., Smith K., Langi V., Edgar M. (2013). The genus Pseudo-nitzschia (Bacillariophyceae) in New Zealand: Analysis of the last decade’s monitoring data. New Zealand J. Mar. Freshw. Res..

[65] Amato A., Lüdeking A., Kooistra W.H. (2010). Intracellular domoic acid production in Pseudo-nitzschia multistriata isolated from the Gulf of Naples (Tyrrhenian Sea, Italy). Toxicon.

[66] Oyarzún D., Brierley C.M. (2019). The future of coastal upwelling in the Humboldt current from model projections. Clim. Dyn..

[67] Brink K., Halpern D., Huyer A., Smith R. (1983). The physical environment of the Peruvian upwelling system. Prog. Oceanogr..

[68] Suárez-Isla B.A., López A., Hernández C., Clement A., Guzmán L., Sar E.A., Ferrario M.E., Reguera B. (2002). Impacto económico de las floraciones de microalgas nocivas en Chile y datos recientes sobre la ocurrencia de veneno amnésico de los mariscos. Floraciones Algales Nocivas en el Cono Sur Americano, Inst. Esp. Oceanogr. Madrid, España. Floraciones Algales Nocivas en el Cono Sur Americano.

[69] Lopez-Rivera A., Pinto M., Insinilla A., Suarez Isla B., Uribe E., Alvarez G., Lehane M., Furey A., James K.J. (2009). The occurrence of domoic acid linked to a toxic diatom bloom in a new potential vector: The tunicate Pyura chilensis (piure). Toxicon.

[70] Álvarez G., Rengel J., Araya M., Álvarez F., Pino R., Uribe E., Díaz P.A., Rossignoli A.E., López-Rivera A., Blanco J. (2020). Rapid Domoic Acid Depuration in the Scallop Argopecten purpuratus and Its Transfer from the Digestive Gland to Other Organs. Toxins.

[71] National Fisheries Health Organization of Perú-Organismo Nacional de Sanidad Pesquera(SANIPES). https://www.sanipes.gob.pe/web/index.php/es/fitoplancton.

[72] Guillard R.R.L., Hargraves P.E. (1993). Stichochrysis immobilis is a diatom, not a chrysophyte. Phycologia.

[73] Hasle G.R., Sournia A. (1978). The Inverted-Microscope Method.

[74] Lundholm N., Hasle G.R., Fryxell G.A., Hargraves P.E. (2002). Morphology, phylogeny and taxonomy of species within the Pseudo-nitzschia americana complex (Bacillariophyceae) with descriptions of two new species, Pseudo-nitzschia brasiliana and Pseudo-nitzschia linea. Phycologia.

[75] Doyle J.J., Doyle J.L. (1987). A rapid DNA isolation procedure for small quantities of fresh leaf tissue. Phytochem. Bull..

[76] White T.J., Bruns T., Lee S., Taylor J. (1990). Amplification and direct sequencing of fungal ribosomal RNA genes for phylogenetics. Pcr Protoc. A Guide Methods Appl..

[77] Hall T.A. (1999). BioEdit: A user-friendly biological sequence alignment editor and analysis program for Windows 95/98/NT. Nucleic Acids Symp. Ser..

[78] Kumar S., Stecher G., Tamura K. (2016). MEGA7: Molecular evolutionary genetics analysis version 7.0 for bigger datasets. Mol. Biol. Evol..

[79] Darriba D., Taboada G.L., Doallo R., Posada D. (2012). jModelTest 2: More models, new heuristics and parallel computing. Nat. Methods.

[80] Stamatakis A. (2014). RAxML version 8: A tool for phylogenetic analysis and post-analysis of large phylogenies. Bioinformatics.

[81] Silvestro D., Michalak I. (2012). raxmlGUI: A graphical front-end for RAxML. Org. Divers. Evol..

[82] Ronquist F., Teslenko M., Van Der Mark P., Ayres D.L., Darling A., Höhna S., Larget B., Liu L., Suchard M.A., Huelsenbeck J.P. (2012). MrBayes 3.2: Efficient Bayesian phylogenetic inference and model choice across a large model space. Syst. Biol..

[83] de la Iglesia P., Gimenez G., Diogene J. (2008). Determination of dissolved domoic acid in seawater with reversed-phase extraction disks and rapid resolution liquid chromatography tandem mass spectrometry with head-column trapping. J. Chromatogr. A.

