# Supplementary material for: First Report of Domoic Acid Production from Pseudo-nitzschia multistriata in Paracas Bay (Peru)
Source: Toxins (Basel). 2021 Jun 9;13(6):408. doi: 10.3390/toxins13060408 (PMC8226791; doi:10.3390/toxins13060408)
Supplement: Supplementary file 1 [file toxins-13-00408-s001.zip › toxins-1237815-supplementary.pdf]

Supplementary Materials: First Report of Domoic Acid Production from *Pseudo-nitzschia multistriata* in Paracas Bay (Peru)

Cecil Tenorio, Gonzalo Álvarez, Sonia Quijano-Scheggia, Melissa Perez-Alania, Natalia Arakaki, Michael Araya, Francisco Álvarez, Juan Blanco and Eduardo Uribe

Table S1. Accurate mass and mass deviation (ppm) of domoic acid and its main fragments.

Ion	Theoretical Mass	Experimental Mass	Delta (ppm)
$[M+H]^+$	312.1447	312.1449	0.64
$[M+H-H_2O]^+$	294.1342	294.1332	−3.39
$[M+H-CH_2O_2]^+$	266.1392	266.1384	−3.00
$[M+H-CH_4O_3]^+$	248.1287	248.1277	−4.03
$[M+H-C_2H_4O_4]^+$	220.1338	220.1329	−4.09

Toxins **2021**, *13*, 408. https://doi.org/10.3390/10.3390/toxins13060408 www.mdpi.com/journal/toxins

Table S2. Comparison of morphometric data between Peruvian strain of *Pseudo-nitzschia multistriata* with strains obtained from different locations around the world.

Apical Axis (µm) Length	Transapical Axis (µm) Width	Fibulae /10 µm	Stria /10 µm	Rows of Poroids	Poroids /1 µm	Band Striae /10 µm	VC/ Stria /10 µm	References
40–48	2.3–3.23	24–28	40–42	2–3	8–13			Paracas Bay, Peru
55–65	3.1–3.6	23–26	37–42	2 (rarely 1 or 3)	5–6			Fukukoka Bay, Japan [1]
44–78	2.8–4	22–29	34–42	2–3	9–13		45–50	Western North Pacific, Peter the Great Bay, Sea of Japan in the coastal waters of Sakhalin Island, Sea of Okhotsk [2]
	2.5–3.8	22–27	37–44		10–12			Sea of Japan [3]
47–53	2.3–3.2	24–29		2	9–12			Tokyo bay [4]
45–78	2.3–4	25–29	32–40	2	9–12		45–46	Amursky Bay, Sea of Japan [5]
37–53	3.4–3.8	25–29		2	10–12			Guangdong coastal waters, South China Sea [6]
58–60	2.5	22–23		2–3	11			Vietnam [7]
43–91	1.4–2.5	23–25	38–40	2–3	11–12			Coasts of Malaysia [8]
	3.3–3.8	26–30	38–40	2 (3)	12			Singapore [9]
38–50	2.5–4	23–32	37–44	2 (rarely 1 or 3)	11–13			Tunisia [10,11]
36–50	2.7–4.1	22–26	36–42	2	9–10			Catalan coast (NW Mediterra-nean) [12,13]
51–70	2.7–3	24–26	36–39	2–3	10–11			Channels of Ria de Aveiro, Portugal[14]
–	2.8–3.6	24–28	38–45	2 (rarely 3)	10–15	48–51		Greek coastal waters [15]
60.2–70.3	3.3–3.8	26–30	38–40	2(3)	12			M'diq Bay, Morocco, Mediter-ranean Sea [16]
38–50	2.5–4	23–32	37–44	2 (rarely 1 or 3)	11–13			Gulf of Naples (Italy, Medi-terranean Sea) [17].
38–50	2.8–3.8	23–28	37–46	2	11–12			Gulf of Naples (Mediterra-nean sea) [18]
35–75	3–3.5	24–26	38–43					Gulf Naples [19]
30–60	2.2–4.6	24–28	34–45	2	7–13			Gulf of Trieste, Northern Adriatic Sea [20]
40.6–42.8	3–3.3							Western Adriatic Sea [21]
59–63	2.5–3	30	40	2 (rarely 3)	12			New Zeeland [22]
26.3–63.7	2.2.–3.9	20–30		2–3	11–12	55–60	2 × 2	Coastal waters of south–east-ern, Australia [23]
52–63	2.5–3.3	25–28	35–40	1–2	11–13			Southern cone of South America [24]
50–57.3	2.7–3.6	23–25	37–40	2	9–10			Manzanillo and Santiago Bay [25]

Table S3. List of sequences of *Pseudo-nitzschia*, *Fragilariopsis* and *Nitzschia* strains included in the molecular analysis. Species, locality, strain code and GenBank accession numbers for the ITS2 gene marker.

Species	Locality	Strain Code	GenBank Accession Number/Sequence Code
Pseudo-nitzschia multistriata	Paracas bay, Peru	IMP-BG 440	**MZ312514**
Fragilariopsis cylindricus	Gulf of St. Lawrence, Anticosti gyre, 49º43´N, 66º15´W, Artic	F_cyl	EF660055
Fragilariopsis nana	49°S, 2°E, Antartic	PA plate12A4	EF660060
Fragilariopsis nana	Ross Sea, 74°0′2′′S 140°0′76′′W, Ice, Antarctic	2-E-F	EF660058
Fragilariopsis nana	Weddell Sea, ice, Antartica	ErikaFcyl	EF660059
Fragilariopsis sp.	NL-2010 Twin Harbor, Washington, USA	Rita9	GU170665
P. heimii/P. subpacifica	Bilbao estuary (43°35′ N, 3°05′ W), Spain	Ner-1D	KC409107
Pseudo-nitzschia abrensis	Bay of Biscay, Atlantic Ocean	NerJ3	KC409109
Pseudo-nitzschia abrensis	Bay of Biscay, Atlantic Ocean	NerJ2	KC409108
Pseudo-nitzschia abrensis	Krokop, Miri, Sarawak, Malaysia	Pnmi19	KR021327
Pseudo-nitzschia abrensis	Rait, Miri, Sarawak, Malaysia	Pnmi168	KR021324
Pseudo-nitzschia americana		Pseud_amer	
Pseudo-nitzschia americana	France	Kervel	EU523099
Pseudo-nitzschia arctica	Beaufort Sea, Canada	RCC2002	KT808253
Pseudo-nitzschia arctica	Beaufort Sea, Canada	RCC2005	KT808255
Pseudo-nitzschia arenysensis	Nervion River Estuary, Spain	NerD1	GQ228393
Pseudo-nitzschia arenysensis	Gulf of Naples, Italy, Mediterranean Sea	AL-24	DQ813830
Pseudo-nitzschia arenysensis	Arenys, Spain	ICMB129	EU367951
Pseudo-nitzschia australis	station LY1 (Lynn of Lorne), western Scottish waters, Scotland, UK	PLYSt19A	AY452527
Pseudo-nitzschia australis	Monterey Bay, California, USA	Au43	DQ062661
Pseudo-nitzschia australis	Santa Cruz Wharf, California, USA	Pn15	KC329501
Pseudo-nitzschia batesiana	Teluk Batik, Perak, Malaysia	PnTb19	KC147514
Pseudo-nitzschia batesiana	Marina Bay, Miri, Sarawak, Malaysia	Pnmi02	KR021328
Pseudo-nitzschia batesiana	Miri, Sarawak, Malaysia	PnMi32	KX572953
Pseudo-nitzschia bipertita	Miri, Sarawak, Malaysia	Pnmi13	KR021320
Pseudo-nitzschia bipertita	Miri, Sarawak, Malaysia	Pnmi04	KR021318
Pseudo-nitzschia bipertita	Miri, Sarawak, Malaysia	Pnmi18	KR021319
Pseudo-nitzschia brasiliana	Samariang Batu, Sarawak, Malaysia	PnSm07	HQ111404
Pseudo-nitzschia brasiliana	Kota Kinabalu, Sabah, Malaysia	PnKk31	JN252429
Pseudo-nitzschia bucculenta	Namibia	L2.6	MH376339
Pseudo-nitzschia bucculenta	Namibia	L1.3	MH376341
Pseudo-nitzschia bucculenta	Namibia	L1.1	MH376340

Pseudo-nitzschia caciantha	Do Son, North Vietnam	DS2	AY257856
Pseudo-nitzschia caciantha	Near Tuxpam, Mexico	Mex20	AY257861
Pseudo-nitzschia caciantha	Sibu Laut, Sarawak, Malaysia	PnSL03	KF482055
Pseudo-nitzschia caciantha	Sibu Laut, Sarawak, Malaysia	PnSL05	KF482056
Pseudo-nitzschia caciantha	Gulf of Naples, Italy, Mediterranean Sea	AL-56	DQ813834
Pseudo-nitzschia calliantha	Gulf of Naples, Italy, Mediterranean Sea	AL-112	DQ813841
Pseudo-nitzschia cf. *subpacifica*	Limens, Spain	Limens8	AY257859
Pseudo-nitzschia cf. *subpacifica*	Costa Nova, Portugal	P-28	AY257858
Pseudo-nitzschia circumpora	Santubong, Sarawak, Malaysia	PnSb58	JN252430
Pseudo-nitzschia cuspidata	Tenerife, Canary Islands, Spain	Tenerife8	AY257853
Pseudo-nitzschia cuspidata	Washington, USA	NWFSC189	KX572955
Pseudo-nitzschia cuspidata	Washington, USA	NWFSC190	JN091757
Pseudo-nitzschia cuspidate	St. John Island, Singapore	PnSg10	KX572957
Pseudo-nitzschia cuspidata	Near Tuxpam, Mexico	Mex12	AY257852
Pseudo-nitzschia cuspidata	Bondi Beach, Sydney, Australia	Sydney1	AY257862
Pseudo-nitzschia cuspidata	Gulf of Naples, Italy, Mediterranean Sea	AL-17	DQ813827
Pseudo-nitzschia decipiens	Kota Kinabalu, Sabah, Malaysia	PnKk38	KP337355
Pseudo-nitzschia decipiens	Tuxpam, Gulf of Mexico, Mexico	Mex13	DQ336156
Pseudo-nitzschia decipiens	Canary Islands, Spain	GranCan4-1	DQ336157
Pseudo-nitzschia delicatissima	Hobart, Tasmania, Australia	Tasm_10	AY257848
Pseudo-nitzschia delicatissima	Ràpita, Catalunya, Spain	ICMB-102	EU478793
Pseudo-nitzschia delicatissima	Gulf of Naples, Italy, Mediterranean Sea	AL-22	DQ813829
Pseudo-nitzschia dolorosa	Monterey Bay, California, USA	Calif1	DQ336152
Pseudo-nitzschia dolorosa	Gulf of Naples, Italy, Mediterranean Sea	AL-59	DQ813835
Pseudo-nitzschia fraudulenta	Limens, Spain	Limens1	AY257840
Pseudo-nitzschia fraudulenta	Arenys, Cataluña, Spain	ICMB-105 (AR3)	DQ990366
Pseudo-nitzschia fraudulenta	French Coast	F10	EU523102
Pseudo-nitzschia fryxelliana	Washington State, USA	NWFSC41	JN050288
Pseudo-nitzschia fryxelliana	Washington State, USA	NWFSC42	JN050287
Pseudo-nitzschia fukuyoi	Marina Bay, Miri, Sarawak, Malaysia	Pnmi158	KR021317
Pseudo-nitzschia fukuyoi	Kota Kinabalu, Sabah, Malaysia	PnKk36	KC147515
Pseudo-nitzschia fukuyoi	Teluk Batik, Perak, Malaysia	PnTb25	KC147516
Pseudo-nitzschia galaxiae	Near Tuxpam, Mexico	Mex23	AY257850
Pseudo-nitzschia galaxiae	Greece	10_4A3	JF714915
Pseudo-nitzschia galaxiae	P. Olimpic, Catalunya, Spain	ICMB173	EU327368
Pseudo-nitzschia galaxiae	Australia	Sydney4	DQ336158
Pseudo-nitzschia granii	Ocean Station Papa (50 N, 145 W)	UBC100	EU051654

Pseudo-nitzschia granii	Cruise MALINA, sampling station PAC060709A, North East Pacific	RCC2273	KT948061
Pseudo-nitzschia hasleana	Washington State, USA	NWFSC252	JN085962
Pseudo-nitzschia hasleana	Hawkesbury River (-33°33′S, 151°18′E), Australia	HAWK3/1	KC017450
Pseudo-nitzschia heimii		NWFSC205	JN091762
Pseudo-nitzschia inflatula	Phuket, Thailand	no7	DQ329204
Pseudo-nitzschia kodamae	Port Dickson, Negeri Sembilan, Malaysia	PnPd26	KF482050
Pseudo-nitzschia kodamae	Port Dickson, Negeri Sembilan, Malaysia	PnPd31	KF482051
Pseudo-nitzschia limii	Rait, Miri, Sarawak, Malaysia	Pnmi06	KR021313
Pseudo-nitzschia limii	Rait, Miri, Sarawak, Malaysia	Pnmi16	KR021311
Pseudo-nitzschia lundholmiae	Teluk Batik, Perak, Malaysia	PnTb10	KC147523
Pseudo-nitzschia lundholmiae	Krokop, Miri, Sarawak, Malaysia	Pnmi28	KR021316
Pseudo-nitzschia lundholmiae	Teluk Batik, Perak, Malacca Strait, Malaysia	PnTb01	KC147522
Pseudo-nitzschia mannii	Pesaro, Adriatic Sea, Italy	CBA56	HE650977
Pseudo-nitzschia mannii	Gulf of Naples, Italy, Mediterranean Sea	AL-101	DQ813839
Pseudo-nitzschia mannii	Thermaikos Gulf, Greece	(08)10A2	JF714905
Pseudo-nitzschia micropora	Van Phong Bay, Vietnam	VPB_B3	AY257847
Pseudo-nitzschia micropora	Kota Kinabalu, Sabah, Malaysia	PnKk14	JN252422
Pseudo-nitzschia multiseries	Monterey Bay, California, USA	Mu3	AY257844
Pseudo-nitzschia multiseries	Ofunato Bay, Japan	OFPm984	DQ062664
Pseudo-nitzschia multistriata	Tarragona, Catalonia, Spain	ICMB-113(CM1)	DQ990367
Pseudo-nitzschia multistriata	Port hacking, New South Wales, Australia	PH25C	KC017470
Pseudo-nitzschia multistriata	Tarragona, Catalonia, Spain	ICMB-115 (CM3)	DQ990369
Pseudo-nitzschia multistriata	Gulf of Naples, Italy	A/B3	EF636680
Pseudo-nitzschia multistriata	Rait, Miri, Sarawak, Malaysia	Pnmi07	KR021314
Pseudo-nitzschia multistriata	43°12'00'' N, 131°54'50'' E; Northwestern Sea of Japan	MBRU_PMS-11	KT247441
Pseudo-nitzschia multistriata	Chin Hae Bay, South Korea	KoreaA	AY257843
Pseudo-nitzschia multistriata	Thermaikos Gulf, Greece	(07)12A2	JF714926
Pseudo-nitzschia multistriata	Gulf of Naples, Italy	B5	EF636677
Pseudo-nitzschia multistriata	Taiwan Strait (Station B1), China	MC4173	MK411976
Pseudo-nitzschia multistriata	Aveiro lagoon, Portugal	Alfa 5	EU684237
Pseudo-nitzschia cf. *obtusa*	Tromsø, Norway	T5	DQ062667
Pseudo-nitzschia plurisecta	Bay of Biscay, Spain, Atlantic Ocean	Ner-G4	KC409088
Pseudo-nitzschia plurisecta	Bay of Biscay, Spain, Atlantic Ocean	Ner-J6	KC409091
Pseudo-nitzschia plurisecta	Bay of Biscay, Spain, Atlantic Ocean	Ner-F1	KC409089

Pseudo-nitzschia plurisecta	Gulf of Maine	En345-153B5	KF006829
Pseudo-nitzschia plurisecta	Tasmania, Australia	Hobart5	AY257851
Pseudo-nitzschia pseudodelica-tissima	Thermaikos Gulf, Greece	9A1	FJ859050
Pseudo-nitzschia pseudodelica-tissima	Gulf of Naples, Italy, Mediterra-nean Sea	AL-15	DQ813826
Pseudo-nitzschia pseudodelica-tissima	Nervion River Estuary, Bay of Biscay, Northern Spain	Ner_D5	GQ228392
Pseudo-nitzschia pungens	Santubong, Sarawak, Malaysia	PnSb44	HQ111413
Pseudo-nitzschia pungens	MuaraTebas, Sarawak, Malaysia	PnMt45	HQ111412
Pseudo-nitzschia sabit	Manzanillo Bay, Colima Mexico	Ps147	KP288507
Pseudo-nitzschia sabit	Port Dickson, Negeri Sembilan, Malaysia	PnPd57	KM400610
Pseudo-nitzschia sabit	Port Dickson, Negeri Sembilan, Malaysia	PnPd68	KM400604
Pseudo-nitzschia sabit	Manzanillo Bay, Colima Mexico	Ps102	KP288506
Pseudo-nitzschia seriata	station LY1 (Lynn of Lorne), western Scottish waters, Scot-land, UK	PLYSt52B	AY452524
Pseudo-nitzschia seriata	Nissum Bredning, Denmark	Nissum3	AY257841
Pseudo-nitzschia simulans	Taiwan Strait, East China Sea	MC984	MF374772
Pseudo-nitzschia simulans	Qingdao, Yellow Sea	MC282	MF374770
Pseudo-nitzschia simulans	Daya Bay, South China Sea	MC281	MF374769
Pseudo-nitzschia simulans	Wanshan Island, South China Sea	MC940	MF374771
Pseudo-nitzschia subcurvata	Ross Sea, Antarctic	1-F	DQ329205
Pseudo-nitzschia subfraudu-lenta	Krokop, Miri, Sarawak, Malaysia	Pnmi71	KR021299
Pseudo-nitzschia subfraudu-lenta	Marina Bay, Miri, Sarawak, Ma-laysia	Pnmi170	KR021298
Pseudo-nitzschia subfraudu-lenta	Thermaikos Gulf, Greece	(08)8A3	JF714929
Pseudo-nitzschia subpacifica	South Korea	HY32E1	LC194952
Pseudo-nitzschia turgidula	North East Pacific Ocean	NWFSC220	JN091764
Pseudo-nitzschia turgiduloides	Ross Sea, Antarctic	3-19	AY257839
Nitzschia longissima	Shilaoren Bay, China	33	KJ671772

Bolded sequence indicates the sequence contributed from this study.

References

1. Takano, H. Marine diatom Nitzschia multistriata sp. nov. common at inlets of southern Japan. *Diatom* **1993**, *8*, 39–41.
2. Stonik, I.V.; Orlova, T.Y.; Lundholm, N. Diversity of Pseudo-nitzschia H. Peragallo from the western North Pacific. *Diatom Research* **2011**, *26*, 121–134, doi:10.1080/0269249x.2011.573706.
3. Stonik, I.; Isaeva, M.; Aizdaicher, N.; Balakirev, E.; Ayala, F. Morphological and genetic identification of Pseudo-nitzschia H. Peragallo, 1900 (Bacillariophyta) from the Sea of Japan. Russ. *J. Mar. Biol.* **2018**, *44*, 192–201, doi:10.1134/S1063074018030100.
4. Yap-Dejeto, L.O.; Takuo & Nagahama, Y. & Fukuyo, Yasuwo. Observations of eleven Pseudo nitzschia species in Tokyo Bay, Japan. *Merine* **2010**, *48*, 1–16.

5. Orlova, T.Y.; Stonik, I.V.; Aizdaicher, N.A.; Bates, S.S.; Leger, C.; Fehling, J. Toxicity, morphology and distribution of Pseudo-nitzschia calliantha, P. multistriata and P. multiseries (Bacillariophyta) from the northwestern Sea of Japan. *Bot. Mar.* **2008**, *51*, 297–306, doi:10.1515/BOT.2008.035.
6. Lü, S.; Li, Y.; Lundholm, N.; Ma, Y.; Ho, K. Diversity, taxonomy and biogeographical distribution of the genus Pseudo-nitzschia (Bacillariophyceae) in Guangdong coastal waters, South China Sea. *Nova Hedwig.* **2012**, *95*, 123–152, doi:10.1127/0029-5035/2012/0046.
7. Larsen, J.; Lam, N.N. Potentially toxic microalgae of Vietnamese waters. *Opera Bot.* **2004**, 5–216.
8. Teng, S.T.; Leaw, C.P.; Lim, H.C.; Lim, P.T. The genus Pseudo-nitzschia (Bacillariophyceae) in Malaysia, including new records and a key to species inferred from morphology-based phylogeny. *Bot. Mar.* **2013**, *56*, 375–398, doi:10.1515/bot-2012-0194.
9. Tan, T.H.; Leaw, C.P.; Leong, S.C.Y.; Lim, L.P.; Chew, S.M.; Teng, S.T.; Lim, P.T. Marine micro-phytoplankton of Singapore, with a review of harmful microalgae in the region. *Raffles Bull. Zool.* **2016**.
10. Sahraoui, I.; Grami, B.; Bates, S.S.; Bouchouicha, D.; Chikhaoui, M.A.; Mabrouk, H.H.; Hlaili, A.S. Response of potentially toxic Pseudo-nitzschia (Bacillariophyceae) populations and domoic acid to environmental conditions in a eutrophied, SW Mediterranean coastal lagoon (Tunisia). *Estuar. Coast. Shelf Sci.* **2012**, *102*, 95–104, doi:10.1016/j.ecss.2012.03.018.
11. Hlaili, A.S.; Khalifa, I.S.; Bouchouicha-Smida, D.; Garali, S.M.; Ksouri, J.; Chalghaf, M.; Bates, S.S.; Lundholm, N.; Kooistra, W.H.; de la Iglesia, P. Toxic and potentially toxic diatom blooms in Tunisian (SW Mediterranean) waters: Review of ten years of investigations. *Adv. Environ. Res.* **2016**, *48*, 51–69.
12. Quijano-Sheggia, S.; Garcés, E.; Sampedro, N.; Van Lenning, K.; Flo Arcas, E.; Andree, K.; Fortuño Alós, J.M.; Camp, J. Identification and characterisation of the dominant Pseudo-nitzschia species (Bacillariophyceae) along the NE Spanish coast (Catalonia, NW Mediterranean). *Sci. Mar.* **2008**, *72*, 343–359.
13. Quijano-Scheggia, S.; Garcés, E.; Andree, K.B.; De la Iglesia, P.; Diogène, J.; Fortuño, J.M.; Camp, J. Pseudo-nitzschia species on the Catalan coast: Characterization and contribution to the current knowledge of the distribution of this genus in the Mediterranean Sea. *Sci. Mar.* **2010**, *74*, 395–410, doi:10.3989/scimar.2010.74n2395.
14. Churro, C.I.; Carreira, C.C.; Rodrigues, F.J.; Craveiro, S.C.; Calado, A.J.; Casteleyn, G.; Lundholm, N. Diversity and abundance of potentially toxic Pseudo-nitzschia Peragallo in Aveiro coastal lagoon, Portugal and description of a new variety, P. pungens var. aveirensis var. nov. *Diatom Res.* **2009**, *24*, 35–62, doi:10.1080/0269249X.2009.9705782.
15. Moschandreou, K.K.; Baxevanis, A.D.; Katikou, P.; Papaefthimiou, D.; Nikolaidis, G.; Abatzopoulos, T.J. Inter- and intra-specific diversity of Pseudo-nitzschia (Bacillariophyceae) in the northeastern Mediterranean. *Eur. J. Phycol.* **2012**, *47*, 321–339, doi:10.1080/09670262.2012.713998.
16. Rijat Leblad, B.; Lundholm, N.; Goux, D.; Veron, B.; Sagou, R.; Taleb, H.; Nhhala, H.; Er-Raioui, H. Pseudo-nitzschia Peragallo (Bacillariophyceae) diversity and domoic acid accumulation in tuberculate cockles and sweet clams in M'diq Bay, Morocco. *Acta Bot. Croat.* **2013**, *72*, 35–47, doi:10.2478/v10184-012-0004-x.
17. Orsini, L.; Sarno, D.; Procaccini, G.; Poletti, R.; Dahlmann, J.; Montresor, M. Toxic Pseudo-nitzschia multistriata (Bacillariophyceae) from the Gulf of Naples: Morphology, toxin analysis and phylogenetic relationships with other Pseudo-nitzschia species. *Eur. J. Phycol.* **2002**, *37*, 247–257, doi:10.1017/s0967026202003608.
18. Sarno, D. Production of domoic acid in another species of Pseudo-nitzschia: P. multistriata in the Gulf of Naples (Mediterranean Sea). *Harmful Algal News* **2000**, *21*, 5.
19. D'Alelio, D.; Amato, A.; Kooistra, W.H.; Procaccini, G.; Casotti, R.; Montresor, M. Internal transcribed spacer polymorphism in Pseudo-nitzschia multistriata (Bacillariophyceae) in the Gulf of Naples: Recent divergence or intraspecific hybridization? *Protist* **2009**, *160*, 9–20, doi:10.1016/j.protis.2008.07.001.
20. Dermastia, T.T.; Cerino, F.; Stanković, D.; Francé, J.; Ramšak, A.; Tušek, M.Ž.; Beran, A.; Natali, V.; Cabrini, M.; Mozetič, P. Ecological time series and integrative taxonomy unveil seasonality and diversity of the toxic diatom Pseudo-nitzschia H. Peragallo in the northern Adriatic Sea. *Harmful Algae* **2020**, *93*, 101773, doi:10.1016/j.hal.2020.101773.
21. Pistocchi, R.; Guerrini, F.; Pezzolesi, L.; Riccardi, M.; Vanucci, S.; Ciminiello, P.; Dell'Aversano, C.; Forino, M.; Fattorusso, E.; Tartaglione, L. Toxin levels and profiles in microalgae from the North-Western Adriatic Sea—15 years of studies on cultured species. *Mar. Drugs* **2012**, *10*, 140–162, doi:10.3390/md10010140.
22. Rhodes, L.L.; Adamson, J.; Scholin, C. Pseudo-nitzschia multistriata(Bacillariophyceae) in New Zealand. *N. Z. J. Mar. Freshw. Res.* **2000**, *34*, 463–467, doi:10.1080/00288330.2000.9516948.

23. Ajani, P.; Murray, S.; Hallegraeff, G.; Lundholm, N.; Gillings, M.; Brett, S.; Armand, L. The diatom genus Pseudo-nitzschia (Bacillariophyceae) in New South Wales, A ustralia: Morphotaxonomy, molecular phylogeny, toxicity, and distribution. *J. Phycol.* **2013**, *49*, 765–785, doi:10.1111/jpy.12087.
24. Méndez, S.M.; Ferrario, M.; Cefarelli, A.O. Description of toxigenic species of the genus Pseudo-nitzschia in coastal waters of Uruguay: Morphology and distribution. *Harmful Algae* **2012**, *19*, 53–60, doi:10.1016/j.hal.2012.05.007.
25. Rivera-Vilarelle, M.; Quijano-Scheggia, S.; Olivos-Ortiz, A.; Gaviño-Rodríguez, J.H.; Castro-Ochoa, F.; Reyes-Herrera, A. The genus Pseudo-nitzschia (Bacillariophyceae) in Manzanillo and Santiago Bays, Colima, Mexico. *Bot. Mar*. **2013**, *56*, 357–373.
